# Exploring hematopoiesis in zebrafish using forward genetic screening

**DOI:** 10.1038/s12276-023-01138-2

**Published:** 2024-01-04

**Authors:** Hyemin Song, Unbeom Shin, Uijeong Nam, Yoonsung Lee

**Affiliations:** 1 Department of Biomedical Sciences, UC San Diego School of Medicine, La Jolla, CA 92093 USA; 2 Salk Institute for Biological Studies, La Jolla, CA 92037 USA; 3 School of Life Sciences, Ulsan National Institute of Science and Technology (UNIST), Ulsan, 44919 Republic of Korea; 4 Department of Biomedical Science and Technology, Kyung Hee University, Seoul, 05278 Republic of Korea; 5 Clinical Research Institute, Kyung Hee University Hospital at Gangdong, School of Medicine, Kyung Hee University, Seoul, 05278 Republic of Korea

**Keywords:** Development, Haematopoiesis, Zebrafish

## Abstract

Zebrafish have emerged as a powerful animal model for investigating the genetic basis of hematopoiesis. Owing to its close genetic and developmental similarities to humans, combined with its rapid reproduction and extensive genomic resources, zebrafish have become a versatile and efficient platform for genetic studies. In particular, the forward genetic screening approach has enabled the unbiased identification of novel genes and pathways related to blood development, from hematopoietic stem cell formation to terminal differentiation. Recent advances in mutant gene mapping have further expanded the scope of forward genetic screening, facilitating the identification of previously unknown genes and pathways relevant to hematopoiesis. In this review, we provide an overview of the zebrafish forward screening approach for hematopoietic gene discovery and highlight the key genes and pathways identified using this method. This review emphasizes the importance of zebrafish as a model system for understanding the genetic basis of hematopoiesis and its associated disorders.

## Zebrafish: a revolutionary model for unravelling hematopoiesis genetics

Zebrafish have been used as a model organism for studying how blood cells are formed since the 1960s due to their high reproduction rate, clear embryos, and diverse gene manipulation techniques. However, the methods to study the genes involved in this process have been limited. This study focuses on the use of forward genetic screening, a technique that introduces random genetic changes and observes their effects, to identify genes involved in hematopoiesis in zebrafish. The researchers used a combination of chemical mutagenesis and next-generation sequencing to identify and analyze these genes. The study identified numerous genes and pathways involved in various aspects of blood development, providing critical insights into the molecular mechanisms underlying hematopoiesis. These findings highlight the important genetic factors influencing blood cell formation and emphasize the crucial role of the caudal hematopoietic tissue niche in regulating blood cell formation.

This summary was initially drafted using artificial intelligence, then revised and fact-checked by the author.

## Introduction

The zebrafish (*Danio rerio*) has been used as a model organism for studying hematopoiesis since the 1960s because of its experimental advantages, including high fecundity, optical transparency of embryos, and diverse gene manipulation techniques^1^. In addition, zebrafish possess evolutionarily conserved hematopoietic genes and regulatory networks, with a comprehensive characterization of all mature blood lineages and intermediates emphasizing its significance in this field^2^. Utilizing diverse genetic approaches in zebrafish, multiple pathways and molecular mechanisms that underlie hematopoiesis have been defined^3^.

Various gene editing approaches have been employed to elucidate the genes associated with hematopoietic processes using zebrafish. The knockdown approach, commonly performed by morpholinos, provides a convenient mechanism to suppress gene expression, allowing rapid insights into gene function^4^. However, this approach has limitations due to concerns about possible off-target effects and inconsistent knockdown efficiencies^5^.

Reverse genetic screening techniques, exemplified by transcription activator-like effector nucleases (TALENs) and clustered regularly interspaced short palindromic repeats (CRISPR)/Cas9, enable the precise targeting and modification of specific genes, serving as powerful tools for validation^6,7^. Notably, the CRISPR system, utilizing guide RNA (gRNA) and the Cas9 enzyme, facilitates efficient gene targeting in terms of cost and time^8^. Over the past decade, the zebrafish model has harnessed the CRISPR system not only to produce knockout (KO) animals but also to execute precise mutagenesis through techniques such as base editing and prime editing^9–11^. While challenges such as off-target effects persist, continuous advancements are being made in these gene-editing techniques.

In addition to reverse genetic methodologies, forward genetic screening is another hallmark of zebrafish research. This unbiased approach provides a platform for identifying novel genes critical for hematopoiesis without predetermined notions. Large-scale forward genetic screening allows the identification of unsuspected players in hematopoiesis, offering insights into the intricate regulatory networks governing this process^12,13^.

To perform a general forward genetic screening in zebrafish, random point mutations are initially induced throughout the genome of adult male zebrafish using a chemical mutagen^14,15^. Inbreeding of heterozygous progeny generated from mutagenized zebrafish produces homozygous mutations in the F3 generation. Through various phenotypic characterizations of hematopoietic events, numerous mutants with hematopoietic defects have been obtained^12,16^. Genetic linkage mapping, along with known polymorphic repeat markers, is critical for identifying the candidate genes responsible for the phenotypes of interest. However, the identification of these causative mutations has been severely limited owing to insufficient genetic markers and laborious work demands^14,17^.

Targeting Induced Local Lesions in Genomes (TILLING) is a hybrid technique that combines elements from both forward and reverse genetic screening. This method involves examining genomic DNA samples from ENU-mutagenized zebrafish to detect mutations in a selected gene. Using sperm from the respective animal, the mutant line is re-established for phenotype characterization^18^. TILLING employs the advantages of forward genetics while also facilitating targeted analysis of specific candidate genes, resulting in the identification of over 150 loss-of-function mutations^19^. Although TILLING provides powerful genetic capabilities, it can be resource intensive, time-consuming, and relatively less effective at inducing complete KOs.

Recently, the advent of next-generation sequencing (NGS) has introduced new genomic and transcriptomic approaches that can sequence nearly the entire zebrafish genome of interest^20,21^. Using a bioinformatics pipeline, thousands of markers across almost any genome of interest can be used to identify causative mutations found by forward screening in a fast, simple, and reliable manner.

In this review, we discuss the application of forward genetic screening in zebrafish to identify significant genes and pathways related to hematopoiesis. We also summarize the key genes discovered through forward genetic approaches and technologies that have helped to dissect the molecular mechanisms of genes involved in hematopoiesis, especially in hematopoietic stem cell (HSC) development. This review provides a comprehensive overview of our current understanding of the molecular mechanisms underlying the development of the hematopoietic system, with a particular focus on zebrafish as a powerful genetic model organism.

## Zebrafish hematopoiesis

**Fig. 1:**
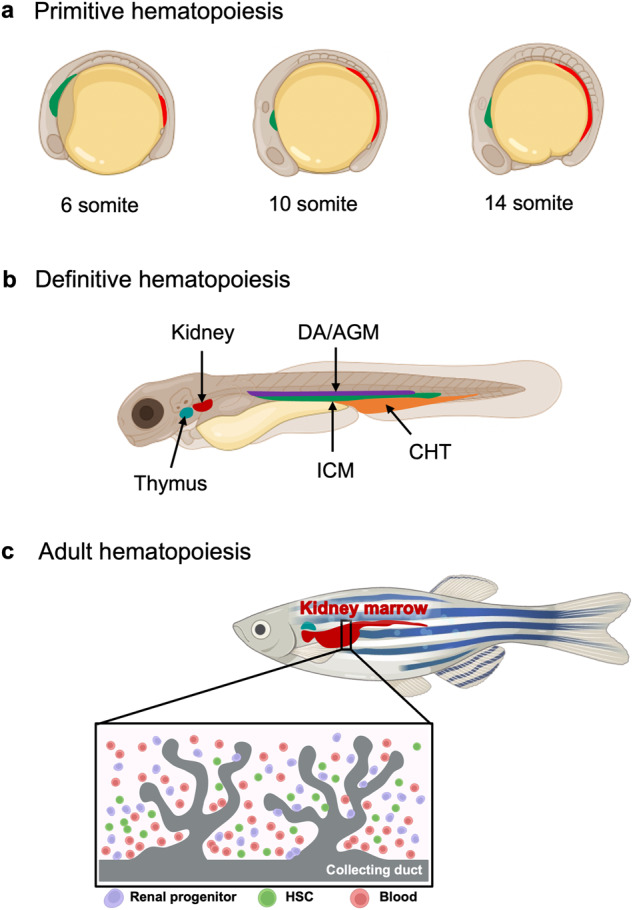
Schematic representation of zebrafish hematopoiesis. **a** Primitive hematopoiesis takes place in two locations, the anterior lateral mesoderm (green) and posterior lateral mesoderm (red), both of which are critical for blood and endothelial formation during the somitogenesis stages. **b** Definitive hematopoietic stem cells (HSCs) arise from the ventral wall of the dorsal aorta (DA) and migrate to the caudal hematopoietic tissue (CHT). HSCs then migrate from the CHT to the kidney, where they undergo lifelong hematopoiesis. HSCs then seed the thymus, where they mature and differentiate into T lymphocytes. **c** Definitive HSCs and other blood cells are distributed within the kidney marrow of adult zebrafish. AGM, aorta gonad mesonephros; ICM, intermediate cellular mass. Figure generated with BioRender.com.

The genetic and regulatory networks of hematopoiesis in zebrafish resemble those in humans. In zebrafish, similar to other vertebrates, hematopoiesis occurs in two distinct waves: primitive and definitive hematopoiesis^22^. Primitive hematopoiesis produces primitive erythroid and myeloid cells by differentiating hemangioblasts, which give rise to both blood cells and vessels^3,23^. It mainly occurs in the anterior and posterior lateral mesoderm, which later becomes the intermediate cell mass where HSCs are congregated (Fig. 1a)^22,24,25^. The generated primitive erythroid and myeloid cells play a crucial role in the transfer of oxygen to tissues and immunity of early embryos and start to circulate throughout the embryos at approximately 24 h post-fertilization (hpf)^26,27^.

Definitive HSCs, which are pluripotent stem cells that give rise to all blood cell lineages, begin to emerge from the ventral wall of the dorsal aorta (DA), a region known as the aorta-gonad-mesonephros, beginning approximately 30 hpf (Fig. 1b)^28–31^. Subsequently, at 2 days post-fertilization (dpf), these cells start migrating toward the caudal hematopoietic tissue (CHT), where they mature and continuously circulate before finally settling in the kidney (Fig. 1b)^32,33^. These definitive HSCs reside in the kidney marrow, which is analogous to the mammalian bone marrow, and produce all the blood lineages necessary for normal blood function from embryonic development to adulthood (Fig. 1c)^22,30,34^. Although many genes and pathways responsible for HSC formation and differentiation have been identified, much remains to be understood. To study the precise steps of HSC specification, differentiation, and maintenance, researchers have utilized zebrafish mutant models generated through various mutagenesis techniques.

## Forward genetic screening for zebrafish hematopoiesis

### N-ethyl-N-nitrosourea (ENU) random mutagenesis

Zebrafish have become a valuable model for large-scale genetic screening because of their ease of genetic manipulation. They are particularly suitable for the unbiased forward screening of specific phenotypes, which is almost exclusively practical among vertebrate models^35^. In forward genetics, random mutations are induced in an organism, and the resulting phenotypes are analyzed to identify mutations that affect the process of interest. Random mutagenesis can be achieved through chemical mutagens such as ENU, physical mutagens such as ionizing radiation, or transposon mutagenesis, all of which randomly induce DNA damage. Among these methods, ENU mutagenesis is a common and efficient chemical mutagenesis method in zebrafish that can induce point mutations randomly in germ cells^36^. In ENU mutagenesis, adult male fish are exposed to water containing ENU for approximately one hour per week over the course of three weeks. The use of anesthetics can increase the survival rate of male fish during this process. ENU mutagenesis induces random single base pair mutations with a high success rate for mutagenic loads in zebrafish premeiotic germ cells^17,37^. Mutagenized zebrafish can exhibit phenotypes that are discretely linked to mutated lesions in a single gene.

### Phenotype screening for zebrafish hematopoiesis

**Fig. 2:**
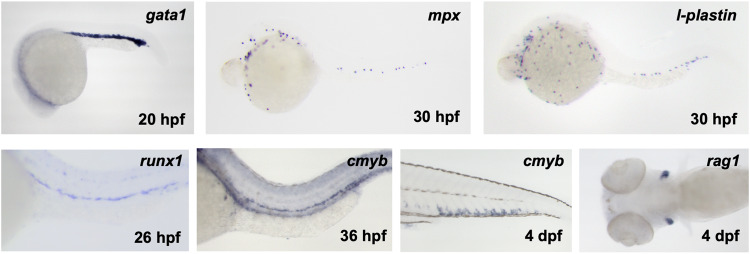
Whole-mount in situ hybridization (WISH) readouts labeling primitive and definitive hematopoiesis. Representative images of WISH using probes labeling primitive erythroid (*gata1*), primitive myeloid (*mpx*, *l-plastin*), definitive HSCs (*runx1*, *cmyb*), and thymocytes (*rag1*) at distinct developmental stages.

Early zebrafish forward screens primarily relied on visually observing live embryos to detect specific morphological defects^38^. Currently, to identify mutants with hematopoietic deficiencies, physically collected mutants displaying distinct morphological defects can be screened for blood development using molecular markers for a given cell or tissue type. Whole-mount in situ hybridization (WISH) is a simple and commonly used approach for detecting the expression site of genes of interest using digoxigenin-labeled antisense RNA probes^39^. Using various hematopoietic markers, the emergence and differentiation of HSCs and other leukocytes can be easily examined in mutant embryos, which can elucidate whether the mutants have hematopoietic developmental defects^40,41^. Moreover, WISH is a useful tool for exploring various tissues and cells important for hematopoiesis, such as primitive erythroid and myeloid cells, as well as definitive HSCs and lymphoid cells (Fig. 2)^42^. Additionally, immunostaining using specific antibodies to observe mutant phenotypes can visualize the development of tissues essential for hematopoiesis processes^41^.

Although WISH and immunostaining are beneficial for increasing the range of phenotypes that can be identified in mutants, these methods require sample fixation, which limits subsequent observations. Tissue-specific transgenic lines have been developed in zebrafish to mark specific cell lineages with fluorescent proteins, enabling the visualization of circulating blood cells in live animals^43,44^. Real-time monitoring of leukocytes facilitates the identification of hematopoietic defects in mutant zebrafish. Large-scale screening using transgenic lines is a feasible approach to assess the development of distinct blood cells and other tissues essential for hematopoiesis. By marking multiple cells and tissues with different fluorescent proteins, transgenic lines facilitate the simultaneous monitoring of the development of blood cells and other hematopoietic tissues^45,46^. Additionally, imaging with transgenic lines yields higher-resolution images compared to those obtained through immunostaining and WISH, leading to more reliable analyses.

## Linkage genetic mapping through NGS

### Traditional linkage analysis

After generating zebrafish mutants and determining their phenotypes through forward screening, the causative genes responsible for the observed phenotype should be identified to further understand the underlying genetic mechanisms. Linkage mapping is a genetic approach used to identify the genomic region responsible for a specific phenotype of interest in mutants^41^. This method involves using known genetic markers on zebrafish chromosomes to map the phenotypes to specific regions of the genome. Genetic markers include a variety of forms, including restriction fragment length polymorphisms, simple sequence length polymorphisms (SSLPs), insertion‒deletion polymorphisms and single-nucleotide polymorphisms (SNPs). These markers are used to track inheritance patterns and map the locations of genes of interest on the chromosome. Once the phenotype of the mutant is linked to a specific region of the genome, the genes responsible for the observed phenotypes can be identified by sequencing the genes in the identified genomic region of the mutants and recovering the altered phenotypes of the mutants by overexpressing the normal gene products of the mutated genes. Although traditional linkage mapping is a powerful tool for identifying novel genes associated with mutants generated via forward genetics, it has several limitations. This method is time-consuming and requires significant resources and expertise. Due to these difficulties, in the first large-scale zebrafish forward genetics study, only 30% of the genes in the mutants could be identified using traditional linkage mapping^14^.

### Whole-genome sequencing (WGS)-oriented linkage mapping

Advances in genetics have led to the development of molecular tools that enable genome-wide analyses to identify the genomic region linked to a mutation. More recently, NGS has become an innovative method for identifying putative genes that may cause the phenotype of interest in zebrafish whole-genome studies^15,47,48^. Although WGS has been widely used to identify candidate genes in *Caenorhabditis elegans* and *Drosophila*^49,50^, it is not a particularly efficient method for zebrafish because of their large and highly polymorphic genome^51^. Thus, sequencing of a single zebrafish genome using WGS is inadequate to accurately distinguish potential causative mutations from other polymorphisms. Instead, bioinformatics approaches have been applied to improve the reliability of sequencing and genetic mapping methods. However, it does not directly address the issue of cost-effectiveness frequently associated with WGS.

**Fig. 3:**
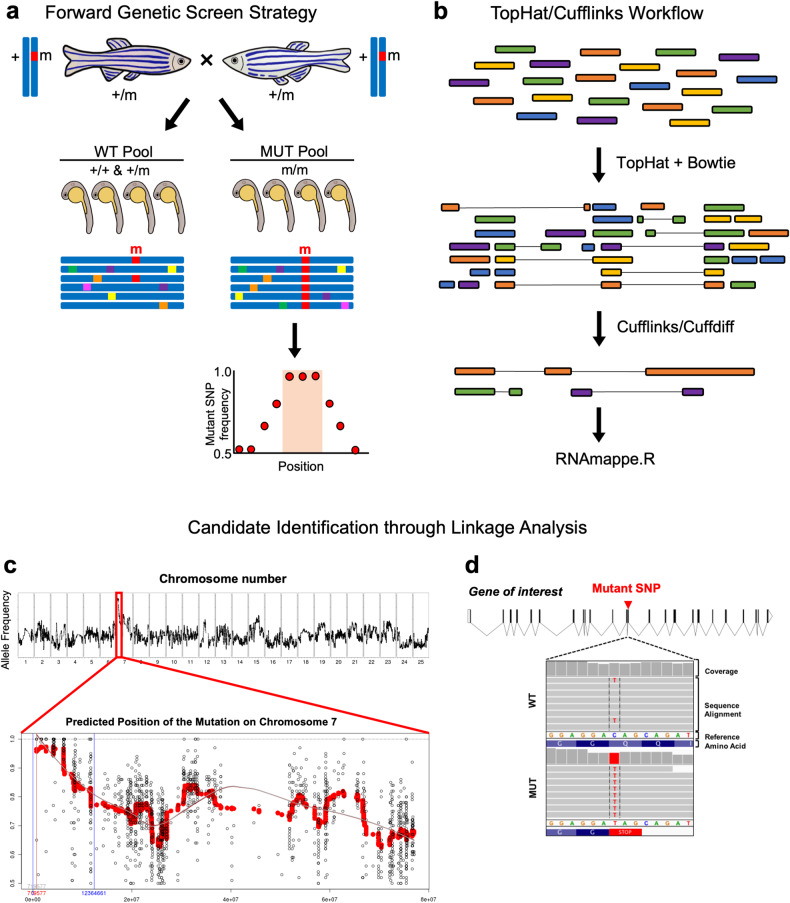
Next-generation sequencing (NGS)-based linkage mapping. **a** Diagram of the forward genetic screening approach. **b** Workflow for transcriptome analysis and identification of differentially expressed genes using TopHat suite and Cufflinks. **c** Alignment of RNA-seq reads to the reference genome using the RNA mapper pipeline, which allows visualization of the whole genome as well as the linked interval on a specific chromosome with high homozygous allele frequency. **d** Position and RNA-seq coverage of SNPs in the gene of interest, which resulted in the introduction of a premature stop codon.

To enhance the process of identifying candidate genes from forward genetic screening, RNA-seq-based bulk segregant analysis has been introduced^52^. This approach is not only cost-effective but also capable of mapping and identifying both genomic regions linked to mutations of interest and mutant genes. A bioinformatics pipeline that employs RNA-seq-based mapping has been developed to identify numerous genes associated with forward genetics. After conducting forward genetic screening, pools of wild-type and mutant zebrafish are separated according to their phenotypes, and their total RNA is processed to prepare RNA-seq samples (Fig. 3a). Following RNA extraction, sequencing libraries are generated for each pool, resulting in multiple samples from several different mutants during sequencing. To analyze the differential expression, RNA sequencing reads from each wild-type or mutant sample are independently aligned and assembled using the TopHat suite^53^. Differential expression is determined, and differentially expressed genes are identified using Cufflinks and the DEGseq R package (Fig. 3b)^54^.

The RNAmapper pipeline is used to input the aligned RNA sequencing datasets and identify the genomic region linked to the mutant phenotype using SNP markers^52^. When forward genetics yields a recessive mutation, the region linked to the mutation exhibits homozygosity. Consequently, a significant peak, indicating a high frequency of the mutant allele at and near the mutation site close to 1, is detected in the linked position of specific chromosomes (Fig. 3c). Once a linkage region is identified, all SNPs within the region are determined from the RNA-seq data of the mutant for candidate SNP filtering. The Ensembl Variant Effect Predictor tool is used to verify whether these candidate SNPs result in nonsynonymous changes^55^. Using the Integrative Genomics Viewer, each SNP is visually observed to ensure that the wild-type and mutant SNPs are segregated appropriately (Fig. 3d)^56^. Ideally, the putative mutant SNP is expected to be present in 100% of the mutants and 33% of the sibling wild-type embryos because of the presence of heterozygous siblings in the pool. Further confirmation is performed using Sanger sequencing to ensure that the candidate SNPs are present in all the mutants.

Overall, RNA-seq-based linkage mapping greatly enhances the identification of a selected number of high-priority candidate mutations associated with phenotypic traits within the linked region. The integration of computational methods has further accelerated and facilitated the reliable and cost-effective identification of causative mutations from forward genetic screens. These ongoing developments and refinements in genetic mapping methods are driving advancements that are improving and reinforcing the knowledge of vertebrate embryogenesis and human disease.

## Primitive hematopoietic genes identified via forward genetics

Over the past 30 years, forward genetic screening studies using zebrafish have identified numerous genes and elucidated the genetic mechanisms involved in hematopoiesis. This section provides examples of the studies that have elucidated the specific roles of genes identified by forward screening in distinct types of hematopoiesis during zebrafish embryogenesis (Table 1).

**Table 1:** Genes involved in hematopoiesis identified using forward genetic screening.

Hematopoietic process	Mutant name	Gene	Phenotype	Readout for screening	Biological character of the mutants	Refs.
Primitive erythropoiesis	*moonshine*	*tif1r*	Bloodless	*gata1* WISH	Apoptosis in erythroid progenitor cells	^12^^,^^58^
	*vlad tepes*	*gata1*	Bloodless	Blood cell count	Inability to bind to the promoter of its target gene	^16^^,^^59^
	*t21384*	*tal1*	Vascular defects	Endothelial alkaline phosphatase activity	Involved in the differentiation of red blood cells	^61^^,^^62^
Primitive myelopoiesis	*mummy*	*dhx8*	No blood circulation	*cbfb* and *l-plastin* WISH	Affects mRNA splicing and cell division during mitosis	^64^
	*moli*	*cebp-a*	Decreased macrophage and neutrophils	Neutral red, Sudan black B staining	Cell cycle arrest of myeloid progenitors	^57^
Early endothelial and blood cells	*cloche*	*PAS-domain-containing bHLH transcription factor*	Blood reduction and vascular defects	GATA-2 expression	Defects in the development of the heart, vasculature and blood	^35^^,^^67^
	*hi2335*	*plcg1*	Bloodless	*cmyb* WISH	Lack of artery specification	^68^
	*hi3714*	*tbx16*	Bloodless	*cmyb* WISH	Lack of axial vascular organization	^68^
	*hi1618*	*hdac1*	Bloodless	*cmyb* WISH	Defect in vessel or artery formation	^68^
HSC specification	*sd45*	*supt16h*	Lack of HSCs	*runx1* WISH	Enlarged hindbrain and curved tail	^69^
HSC maintenance	*rumba*	C2H2 zinc finger protein	Lack of T lymphocytes	*rag1* WISH	Delayed cell cycle	^70^
	*samba*	*has3*	Lack of T lymphocytes	*rag1* WISH	Cell arrested in M phase	^70^
	*lack*	*sae1*	Lack of T lymphocytes	*rag1* WISH	Impaired SUMOylation	^71^
	*116*	*rpc9*	T-cell deficient	*rag1* WISH	p53-dependent apoptosis	^72^
	*ceylon*	*tbl3*	Lack of lymphoid cells	*rag1* WISH	Reduced population of T cells and red blood cells	^40^^,^^73^
	*grechetto*	*cpsf1*	Bloodless	*mpx* WISH	Abnormal polyadenylation of gene needed for HSC development	^74^
	*cas003*	*topbp1*	Decreased HSCs	*cmyb* WISH	p53-dependent apoptosis due to abnormal ATR/Chk1 activation	^75^
	*cas002*	*kri1l*	Decreased HSCs	*cmyb* WISH	Autophagy due to accumulation of unfolded protein activation of PERK	^76^
	*cas008*	*gemin5*	Reduced *myb* expression	*cmyb* WISH	Reduced proliferation of HSCs	^77^
	*smu07*	*slc20a1b*	Bloodless	*βe1-globin* WISH	Cell arrested in G2/M phase	^78^^,^^79^
HSC niche	*cas005*	*itga4*	Decreased HSCs	*cmyb* WISH	Defective interaction between HSC and macrophages	^80^
	*oloca*	*naca*	Decreased neutrophils	Sudan black B staining	Reduced survival of stromal cells	^81^

Erythropoiesis

Primitive hematopoiesis is regulated by a complex interplay of genes, and this area of research is constantly evolving. Nusslein-Volhard et al. conducted a pioneering forward genetic screening study in the 1990s to characterize the phenotypes of mutants associated with primitive hematopoiesis^12,16,57^. Subsequently, mapping identified the relevant genes, and further research was conducted to determine the causes of hematopoietic defects in these mutants^58,59^. For instance, by confirming ENU-induced mutations based on GATA-1/GATA-2 expression and analyzing isolated blood cells, a *moonshine* (*mon*) mutant with a bloodless phenotype in the embryo was obtained^12^. Subsequent investigation identified the *mon* gene as transcriptional intermediary factor 1 gamma (*tif1γ*) through positional cloning using microsatellite markers, revealing that mutations in *tif1*γ trigger apoptosis in erythroid progenitor cells^58^.

In addition to *tif1γ*, the *gata1* gene has also been identified as a primitive erythropoiesis marker through forward genetic screening. The initial mutant, named *vlad tepes* (*vlt*), was isolated by visually observing a decrease in the number of red blood cells^16^. Diploid mapping using centromeric markers confirmed that *vlt* is the *gata1* gene^59^. Further investigation revealed that the mutated form of *gata1* could not bind to the promoter of its target genes, resulting in erythropoietic defects. Another erythropoietic gene identified through forward screening is *tal1*, which was mapped using SSLP markers^60,61^. Initially discovered in a screening study to identify genes involved in blood vessel development, subsequent studies revealed that *tal1* plays a critical role in red blood cell differentiation, with a lack of *tal1* leading to anemia^60,62^. This discovery implies that hemangioblasts, which are precursors of primitive hematopoiesis, can differentiate into endothelial cells. Consequently, mutations affecting blood vessel development can also impact primitive hematopoiesis^3,23^.

Myelopoiesis

Forward genetic screening has also yielded mutants that cause defects in primitive myelopoiesis, which is crucial for early embryonic immunity^23,27^. Liu et al. conducted ENU mutagenesis and screened mutant embryos via WISH using probes for the myeloid cell markers *cbfb* and *l-plastin*^63,64^. A mutant named *mummy* (*mmy*) with reduced expression of both myeloid markers was identified. The *mmy* mutant displayed no blood circulation and a reduction in markers related to erythropoiesis, indicating that both myelopoiesis and erythropoiesis were affected. Subsequent positional cloning of the *mmy* mutant identified the causative mutation in the splicing factor gene *dhx8*^64^.

Another mutant, *moli*, related to primitive myelopoiesis, was generated by ENU mutagenesis and screened using neutral red and Sudan Black to identify macrophages and neutrophils, respectively^57^. These mutants showed a reduction in both macrophages and neutrophils and induction of cell cycle arrest in myeloid progenitor cells. Through linkage mapping using SSLP markers, the gene encoded by the *moli* mutant gene was found to be a bZIP transcription factor gene, *cebpα*, indicating that Cebpα plays an essential role in the maintenance of neutrophils during zebrafish embryogenesis.

## Forward screening for HSC development

Given that blood cell lineages are generated through HSC development, understanding the detailed molecular mechanisms of HSC emergence and differentiation is crucial. Numerous key genes associated with HSC development have been identified through forward genetic approaches involving ENU-induced mutations. For instance, the functional role of *cmyb*, a widely recognized HSC marker, was identified through ENU-induced forward genetic screening^65,66^. Zebrafish embryos carrying *cmyb* mutations show failure of definitive hematopoiesis, indicating the essential role of this gene in regulating HSC development. Additionally, various forward screening studies have provided valuable insights into the function of novel genes related to HSC precursor formation, HSC specification and maintenance, and the regulation of HSC development by environmental signals.

Early endothelial and blood cells

Many genetic aberrations associated with HSC emergence and differentiation have been identified through ENU mutagenesis screening. For example, the *tal1* mutant phenotype exhibited a profound underdevelopment of the vasculature with the loss of not only *gata1*, a primitive erythrocyte marker, but also *runx1*, an early definitive HSC marker^61^. In addition, the *cloche* mutant embryos displayed cardiac abnormalities with a substantial reduction in both blood and vasculature formation^35^. The absence of GATA-2 expression, a key marker for early hematopoietic stem and progenitor cells in intermediate cellular mass (ICM), underscores the indispensable role of *cloche* in HSC generation^26^. Recent studies have revealed that the causative gene for *cloche*, which plays a significant role in both vascular and blood specification, is a PAS domain-containing bHLH transcription factor^67^.

Other mutants, such as *plc*γ*1, tbx16, and hdac1*, manifest defective axial vascular organization, artery specification, and vessel or artery formation, respectively^68^. Notably, all of these mutants exhibit a deficiency in HSC formation, as evidenced by the complete loss of *runx1* expression. These genetic anomalies collectively accentuate the interplay between vascular and hematopoietic morphogenesis. Therefore, the development of the vasculature, including the DA, veins, and arteries, emerges as a fundamental orchestrator of HSC generation from endothelial cells in the ventral wall of the DA. Consequently, ensuring proper vascular morphogenesis is necessary for the initiation and maintenance of HSCs.

HSC specification

Forward genetic approaches have identified genes that specifically impact the formation and specification of definitive HSCs without affecting primitive hematopoiesis and vasculogenesis. Recently, we identified a novel gene, *supt16h*, using forward genetics^69^. The *supt16h* mutants displayed an enlarged hindbrain and a deformed tail at 32 hpf. These traits were linked to the failure of HSC formation and proliferation, and the mutants displayed normal formation of upstream or adjacent tissues and cells, including posterior lateral mesoderm, sclerotome, vasculature, primitive erythrocytes, and primitive leukocytes. Furthermore, the loss of *supt16h* disrupts Notch activation, a pathway that is crucial for HSC emergence and specification. Subsequent investigations revealed that the absence of *supt16h* leads to increased expression of *p53*, which elevates the expression of *phc1*, a Notch repressor gene, resulting in the inhibition of Notch activation and HSC formation. This study demonstrated the significant role of *supt16h* in the Notch pathway and the regulation of HSC development. The study also exemplifies how forward genetic screens can not only identify novel genes of interest but also guide further research on the molecular mechanisms relevant to hematopoietic development.

Expansion and maintenance of HSCs

Through forward genetic screening, numerous novel genes involved in the expansion and maintenance of definitive HSCs have been identified. Three distinct mutants generated by ENU mutagenesis (*rumba*, *samba*, and *tango*) were identified through reduced expression of thymic *rag1*, a definitive hematopoiesis marker. Subsequent positional cloning revealed that *rumba* encodes a novel zinc finger protein, *samba* encodes the mitotic spindle protein Haus3, and *tango* encodes SUMO-activating enzyme subunit 1 (*sae1*)^70,71^. Loss of *rumba* and *haus3* results in defective HSC maintenance due to alterations in the cell cycle profile, whereas loss of *sae1* reduces HSC survival owing to SUMOylation defects. Another ENU screening study using thymic *rag1* expression identified mutated RNA polymerase III component 9 (*rpc9*) in the mutant line with definitive hematopoiesis defects. Loss of *rpc9* leads to a defect in HSC maintenance by inducing p53-dependent apoptosis^72^. The *ceylon* (*cey*) mutants identified through definitive *rag1* WISH exhibit severe defects in HSC maintenance^40,73^. SSLP-based linkage mapping identified the causative mutation in the *cey* mutants within the *tbl3* gene, which is associated with cell cycle regulation. Moreover, *grechetto* mutants were identified by employing the pan-leukocyte marker *mpx* in the CHT at 5 dpf. Positional cloning revealed that cleavage and polyadenylation specificity factor 1 (*cpsf1*) is the mutated gene in *the grechetto* mutant^74^. Further characterization of the mutant suggested that proper mRNA maturation is needed for HSC survival during definitive hematopoiesis.

In addition, the *cmyb* WISH screening method was used to identify multiple genes related to HSC maintenance and expansion. Pan et al. obtained HSC-defective mutants exhibiting reduced *cmyb* expression via ENU mutagenesis and subsequently mapped the mutated genes using bulk segregation analysis along with SSLP markers. Three genes, *topbp1, kri1l*, and *gemin5*, were found to be linked to the mutant phenotypes^75–77^. Further characterization of these mutants showed that TopBP1 is involved in HSC maintenance through regulation of p53-dependent apoptosis in the CHT. Loss of the rRNA maturation factor *kri1l* leads to misfolded proteins in HSCs and induces autophagy via PERK signaling. *gemin5* mutants exhibited a significantly decreased number of HSCs in the CHT due to defective HSC proliferation. Additionally, in a separate study, *slc20a1b* was found to be essential for HSC expansion in the CHT through *βe1-globin* WISH forward screening and subsequent analysis of *cmyb* expression in the CHT^78,79^.

Niche for HSC expansion

Forward genetic studies have provided valuable insights into the crucial roles of niches where HSC expansion and maintenance occur. Utilizing *cmyb* WISH screening, a genetic mutation in integrin alpha 4 (*itga4*) that causes severe defects in definitive hematopoiesis has been identified^80^. Further investigation using photoconvertible transgenic lines revealed that the HSC retention time in the CHT is reduced in the *itga4* mutants. The loss of *vcam1*, a ligand of the Itga4-Itgb1 complex, leads to similar defects in definitive hematopoiesis as those observed in *itga4* mutants. Notably, macrophages expressing *vcam1* emerge as critical players in HSC retention within the CHT niche, highlighting the importance of the niche microenvironment in regulating HSC function^80^.

In another forward genetic study, researchers identified the *oloca* mutant using Sudan Black staining, which exhibited a decreased number of neutrophils compared to wild-type embryos at 3.5 dpf^81^. The *oloca* mutant carries a mutation in the *naca* gene, resulting in a reduced number of HSCs in the CHT, accompanied by abnormal proliferation and differentiation. Further investigations showed that Naca within the stromal niche plays a non-cell autonomous role in facilitating the settlement and formation of HSCs in the CHT. These findings highlight the significant genetic factors influencing HSC expansion and underscore the pivotal role of the CHT niche in regulating HSC function.

## Summary and conclusion

In recent years, zebrafish have emerged as a powerful animal model for studying the genetic basis of hematopoiesis. Various gene editing and analysis techniques, including forward genetic screening, reverse screening, CRISPR-based approaches, and NGS, have been employed to identify genes associated with hematopoiesis and blood disorders. Notably, forward genetic screening, exemplified by methods such as ENU mutagenesis combined with linkage mapping techniques, has proven particularly advantageous in the zebrafish model. This strategy has facilitated the unbiased identification of numerous genes and pathways that regulate various aspects of blood development. In this review, we have explored the diverse genes and pathways involved in hematopoietic processes, providing critical insights into the molecular mechanisms underlying hematopoiesis via forward genetic screening.

As mutant mapping technologies advance and our knowledge of zebrafish genetics expands, we anticipate that this genetic model system will continue to revolutionize our understanding of hematopoiesis and blood disorders. Recent analyses using NGS and genome-wide association studies (GWASs) on extensive databases have highlighted potential blood disease-related genes and their associated pathological mechanisms^82,83^. Building on data-driven studies, zebrafish gene editing via the CRISPR system enhances our understanding of the related disease mechanisms and facilitates the development of disease models^84^. Collectively, these multifaceted gene editing approaches epitomize the utility of the zebrafish model in deciphering the complexities of hematopoiesis and its relevance to human health^3^.
